# Pain Burden in Post-COVID-19 Syndrome following Mild COVID-19 Infection

**DOI:** 10.3390/jcm11030771

**Published:** 2022-01-31

**Authors:** Indre Bileviciute-Ljungar, Jan-Rickard Norrefalk, Kristian Borg

**Affiliations:** 1 Department of Clinical Sciences, Karolinska Institutet, 182 88 Stockholm, Sweden; norrefalk@hotmail.com (J.-R.N.); kristian.borg@ki.se (K.B.); 2 Multidisciplinary Pain Clinic, St Göran Hospital, 112 19 Stockholm, Sweden; 3 Department of Rehabilitation Medicine, Danderyd University Hospital, 171 77 Stockholm, Sweden

**Keywords:** post-COVID-19 syndrome, chronic pain, widespread pain, fibromyalgia, fatigue, post-exertional malaise, comorbidities, medication, quality of life

## Abstract

The global pandemic of SARS-CoV-2 has affected several hundred million people, and many infected people have suffered from a milder initial infection but have never fully recovered. This observational study investigates the pain burden in sufferers of post-COVID-19 syndrome after a milder initial infection. One hundred post-COVID-19 patients filled out questionnaires regarding sociodemographic data, previous comorbidities, present pharmacological treatment, pain intensity and pain localisation. Health-related quality of life, fatigue, emotional status, and insomnia were measured by validated questionnaires. Multiple post-COVID-19 symptoms, including post-exertional malaise, were evaluated by a symptom questionnaire. Among the 100 participants (mean age 44.5 years), 82% were women, 61% had higher education, and 56% were working full or part time. Nine participants reported previous pain or inflammatory conditions. Among the most painful sites were the head/face, chest, lower extremities, and migrating sites. Generalised pain was self-reported by 75 participants and was estimated in 50 participants. Diagnosis of fibromyalgia according to the 2016 criteria was suspected in 40 participants. Subgroup analyses indicated that comorbidities might play a role in the development of pain. In conclusion, a major part of sufferers from post-COVID-19 syndrome develop pain, and in addition to its many disabling symptoms, there is an urgent need for pain management in post-COVID-19 syndrome.

## 1. Introduction

Since the start of December of 2019, the COVID-19 pandemic has affected several hundreds of millions of people all over the world, resulting in over five million deaths and overloaded healthcare systems in many countries. Infections vary between asymptomatic and lethal, and sufferers either recovered at home or were hospitalised. The neurological impact of the SARS-CoV-2 virus has been speculated both in the pathways of respiratory failure [1] as well as other neurological pathways [2]. The acute symptomatology is well known, including respiratory failure, thrombosis, kidney failure, etc. [3,4], but the long-term symptoms are only now starting to bother patients during the subacute (3–6 months after infection) and chronic periods (longer than 6 months after infection). Long COVID or post-COVID-19 syndrome appears both in hospitalised [5] and un-hospitalised patients [6].

The World Health Organisation has appealed to healthcare-givers for the development of post-COVID-19 syndrome as a sequalae of SARS-CoV-2, and it has estimated that approximately 10% of all infected people may suffer from it [7]. According to the WHO, post-COVID-19 syndrome occurs in individuals with a history of probable or confirmed SARS-CoV-2 infection, usually 3 months from the onset of COVID-19 with symptoms that last for at least 2 months and cannot be explained by an alternative diagnosis. Common symptoms include fatigue, shortness of breath, and cognitive dysfunction as well as others that generally have an impact on everyday functioning. Symptoms may be new onset, following initial recovery from an acute COVID-19 episode, or might persist from the initial illness. Symptoms may also fluctuate or relapse over time [7].

Hospitalisation during the acute period makes it possible to follow-up with patients and to identify remaining symptoms such as fatigue, breathing difficulties, cognitive symptoms, persistent musculoskeletal pain, sleeping difficulties, etc. [8,9]. Irrespective of the severity of the initial infection, persistent fatigue seems to be the most bothersome symptom of post-COVID-19 syndrome [10]. During an acute SARS-CoV-2 infection, pain as a symptom is mostly found as a headache, sore throat, and arthralgias/myalgias [11]. A meta-analysis of the prevalence of headaches after COVID-19 infections yielded estimates of approximately 8–15% at 6-month follow-ups [12]. Karaarslan et al. reported that myalgia was still reported by 21% and arthralgia by 22% of patients at 1-month follow-ups after a short hospitalisation (not in the ICU ward) due to COVID-19 infection [13].

Recently, we reported the International Classification of Functioning and Disability (ICF)-based data on body functions and activities/participation from the same population as the present study [14]. In summary, the following body functions were impaired in our participants: energy and drive (98–99%), higher cognitive functions (74–94%), sleep functions (98%), muscle functions (93%), respiratory functions (92%), heart functions (82%), emotional functions (80%), sexual functions (77%), and thermoregulatory functions (68%) [14]. Functional impairments due to pain were reported as (1) pain in one part of the body (90% of participants), (2) pain in multiple body parts (83%), (3) generalized pain (65%), and (4) radiating pain in a segment or region (56%). The aim of the present study was to further explore pain characteristics among other symptoms in post-COVID-19 syndrome after mild infection. The hypotheses of the study were (1) post-COVID-19 sufferers develop a pain burden in addition to other disabling symptoms, and (2) previous comorbidities affect pain characteristics in post-COVID-19 syndrome.

## 2. Materials and Methods

### 2.1. Participants

Participants were recruited by Facebook sites and a stakeholder’s organisation for post-COVID-19 syndrome in Sweden (Svenska covidföreningen). An online announcement briefly describing the study with inclusion and exclusion criteria was published alongside a further link for more detailed information for those who were interested in participating. Thereafter, participants could sign into the online platform BASS at the eHealth Core Facility at Karolinska Institutet using a two-step identification factor. The online platform BASS makes it possible to collect an electronically signed agreement to participate as well as to collect all questionnaires. The data were collected from the end of April to the end of August 2021, and all consecutive participants were considered in the analysis.

The inclusion criteria were (a) COVID-19 infection supported by anamnesis and/or positive tests for COVID-19 virus (PCR) and/or positive immunoglobulin response; (b) age between 18 and 70 years; (b) significantly reduced level (at least 50%) of functioning and activity/participation in daily life as compared to before infection; (c) persistent symptom duration of at least 12 weeks after acute infection; (d) satisfactory management of any co-morbidities; and (e) being able to use the Internet, to complete Internet-based questionnaires, and to participate in a rehabilitation programme delivered through the Internet for a group of a maximum of 25 participants during an 8-week period. The exclusion criteria were (a) unclear onset of symptoms in relation to COVID-19 (for example stress factors, post-traumatic stress disorder, or other types of psychological or somatic trauma in combination with the COVID-19 infection or before it, (b) abuse of alcohol or psychotropic substances, (c) diagnosis of a psychological or somatic condition that requires appropriate treatment (e.g., hypothyroidism; lung, heart, or kidney diseases; psychosis; suicidality; etc.), and (d) ongoing psychological or medical treatments that may interfere with rehabilitation (for example, other psychotherapies or ongoing introduction or adjustment of pharmacological drugs). All data were self-reported by the participants and gathered after recruitment and before randomisation to either treatment or to a control waiting list for a randomised rehabilitation study for post-COVID-19 sufferers.

Because this study was planned as a rehabilitation study, the Internet-collected data were combined with face-to-face meetings online during a rehabilitation programme. Fifty participants had already participated in 8 weeks of multimodal group rehabilitation online with approximately six individual appointments with a team member, including a doctor (the first author, IBL). These weekly appointments were discussed by team members and made it possible to confirm the appropriateness of participation in the study according to the inclusion and exclusion criteria, post-COVID-19 syndrome diagnosis, comorbidities, and pharmacological treatment, including pain-related issues. Although the region one lives in in Sweden was not included in the questionnaire, individual appointments revealed that approximately 30% of the 50 participants came from Stockholm. Therefore, the remainder of participants came from other parts of Sweden thanks to the Internet-based study design.

Participants briefly filled out the sociodemographic data and questionnaires regarding persistent symptoms after COVID-19 infection using BASS. The study is registered on ClinicalTrials.gov, accessed on 27 January 2022 (Identifier: NCT04961333) and was approved by the Swedish Ethical Authorities (Etikprövningsmyndigheten), Dnr. 2020-07216.

### 2.2. Questionnaires

Sociodemographic questions included age, gender, body weight and height, education, socioeconomic status, use of alcohol and tobacco, comorbidities before SARS-CoV-2, and ongoing medication. Comorbidities and medication were indicated in free text and checked that they corresponded to respective diagnoses (for detailed information, see Supplemental Material S1). Preventive hormonal medication, vitamins, and non-prescribed drugs/supplements were not analysed and are not presented here.

Pain measurements. Pain intensity during the last week was measured using an 11-point numeric scale (0 = “no pain” to 10 = “worst imaginable pain”). Pain sites were scored by the presence or absence of pain in 36 body sites and the most painful sites.

The symptom questionnaire contained questions on multiple body symptoms related to COVID-19 infection, graded from “none” to “unbearable” (0–4) (see Supplemental Material S1).

The European Quality of Life Instrument (EQ-5D) captures patients’ perceived state of health and is presented as the EQ5D Index and EQ5D VAS. For the normal Swedish population, EQ5D is 0.85, and the corresponding EQ5D VAS is 85 for people between 35 and 54 years old [15].

The Short-Form 36 (SF-36) is a validated and widely used questionnaire to assess health-related quality of life. Here we report only the subscale Bodily Pain. Values equal to or lower than 73 were considered abnormal for the Bodily Pain dimension [16,17].

The Swedish version of the Multidimensional Fatigue Inventory (MFI-20) covers five dimensions of fatigue [18]. Values for the normal population were chosen as previously reported: general fatigue ≤ 11; physical fatigue ≤ 8; mental fatigue ≤ 9; reduced motivation ≤ 8, and reduced motivation ≤ 7 [19].

The Hospital Anxiety and Depression Scale (HADS) is used to determine the levels of anxiety and depression a person is experiencing [20]. A score of ≥11 is an indication of clinically significant anxiety or depression symptoms [20].

The Patient Health Questionnaire-9 (PHQ-9) is designed to assess depressive symptom severity [21]. The standard cut-off score for screening to identify possible major depression is ≥10 [22].

The Generalised Anxiety Disorder-7 scale (GAD-7) is used to assess anxiety symptom severity. The standard cut-off score for screening to identify possible anxiety disorder is suggested to be ≥10 [23].

The Insomnia Severity Index (ISI) measures insomnia. Values between 8 and 14 indicate some problems with sleep, while values ≥15 indicate moderate sleep disturbance and values >22 indicate clinically significant sleep disturbances [24].

### 2.3. Calculation of Generalised Pain and Fibromyalgia Diagnosis According to the 2016 Criteria

Fibromyalgia diagnosis was made according to the criteria of (1) generalised pain in 4 out of 5 body regions and (2) Widespread Pain Index ≥ 7 and Severity Symptom Scale ≥5 or Widespread Pain Index ≥ 4–6 and Severity Symptom Scale ≥ 9 [25]. More information regarding the scales and the calculation of fibromyalgia diagnosis according to the 2016 criteria is summarized in Supplemental Material 1. The answers from the questionnaires, including previous comorbidities, present symptoms, and ongoing medication, were checked during the face-to-face meetings with the 50 participants who participated in the telemedicine multimodal rehabilitation for 8 weeks.

### 2.4. Statistical Analysis

SPSS version 28 (IBM SPSS Statistics, Armonk, NY, USA).was used for all analyses. Descriptive statistics for nominal data (sociodemographic data, presence/absence of symptoms, pain sites, etc.) are presented as the number of participants per group. Body mass index (BMI) is presented as the mean, standard deviation, and range. Results from the questionnaires EQ5D, SF-36 Bodily Pain, MFI-20, HADS, PHQ-9, GAD-7, and ISI (ordinal data) are presented as mean values, standard deviations, and ranges and as the numbers of participants having abnormal values.

Statistical subgroup analysis was performed using 2-tailed Chi-squared tests for nominal data. For ordinal data (number of drugs and comorbidities), the Mann–Whitney U-test was used for between-group analysis. An independent-samples t-test was used for comparing BMI. A *p*-value lower than 0.05 was considered statistically significant.

## 3. Results

### 3.1. Sociodemographic Characteristics of Participants

Data from 106 consecutive participants were obtained. One participant was excluded due to the exclusion criteria (ongoing rehabilitation due to previous comorbidities), and five participants were excluded due to missing data. Among the 100 included participants, the mean age was 44 years, 82% were female, 88% were born in Sweden, 49% were married, 73% had children, 53% lived in their own houses, 61% had higher education, 56% were working full or part time, 81% had employment, and 19% were seeking a job, studying, or had other financial support during the recruitment period. Regarding benefits from the social security system, 38% were on full or part-time sick-leave, 13% had disability pensions, 3% received unemployment benefits, and 4% had social security contributions. The sociodemographic data are summarized in Table S1.

Regarding harmful habits, only one participant smoked, none consumed un-prescribed narcotics, 54 did not consume alcohol, 15 habitually drank one glass of wine/beer or one unit of alcohol per week, and 12 drank two glasses of wine per week. The maximum amount of six glasses of wine per week was drunk by 1 participant, and 2 participants consumed 1 litre of wine per week.

The period between the start of SARS-CoV-2 infection and completing the questionnaire was a mean of 47 weeks (standard deviation 20 weeks, range 12–83 weeks). Eighty-six percent indicated that they were tested with a PCR laboratory test for SARS-CoV-2, and 46% of the participants reported a positive result. Those who reported a negative PCR-test (40%) or did not leave any answer (14%) were from the first pandemic wave, except for two participants. Eighty-one percent reported that they had been tested with an antibody test for SARS-CoV-2. Only three participants reported either a negative or absent PCR test and an absent antibody test. Among them, one reported having been denied a primary health-care appointment during the first pandemic wave, and two reported having been diagnosed with post-COVID-19 syndrome by clinical symptoms. Most participants (90%) were not hospitalised during the acute infection, while seven (7%) were hospitalised once for 1–3 days, two (2%) were hospitalised twice for 2–5 days, and one participant (1%) was hospitalised for 3 months, including the intensive care unit.

### 3.2. Comorbidities and Medication

Table 1 presents comorbidities before the COVID-19 infection and ongoing medications. Sixty-eight percent of participants reported being completely healthy before the COVID-19 infection, without having had any contact with the healthcare system. Most of the comorbidities before the COVID-19 infection concerned heart, lung, metabolic, inflammatory/pain, and psychiatric comorbidities. Only three participants reported “clear” pain syndromes before the COVID-19 infection, including fibromyalgia (1 participant), Ehlers Danlos syndrome (1 participant), and migraine (1 participant). Regarding medication for pain, 12 participants consumed non-steroid anti-inflammatories or other inflammatory medication, 13 reported that they took tricyclic or tetracyclic antidepressants, 1 consumed opioids (low dose tramadol), 3 consumed paracetamol, 6 took antiepileptics such as gabapentin or pregabalin, and 23 were taking psychiatric drugs. Among the psychiatric drugs, five were prescribed serotonin-norepinephrine reuptake inhibitors (duloxetine or venlafaxine). Sleep medication was taken by 17 participants. Regarding total medication among the analysed drugs, 28 participants were not taking any medication, while 31 participants were taking 1 drug, 18 were taking 2 drugs, 13 were taking 3 drugs, 4 were taking 4 drugs, and 3 were taking 5 drugs. One participant each was taking 6, 7, and 9 drugs. The population had a slight tendency to be overweight with a mean BMI of 26.5, Table 1.

When analysing those who indicated that they were “healthy” before the COVID-19 infection in comparison to those who indicated that they were “unhealthy” before the COVID-19 infection, a statistically significant difference was found regarding medication for asthma, hypothyroidism, and psychiatric disorders (Table 1). “Unhealthy” before COVID-19 participants were taking these medications more frequently. “Unhealthy” before COVID-19 infection persons also had higher BMIs after infection compared to “healthy” persons (Table 1).

### 3.3. Pain Characteristics in Participants

**Figure 1:**
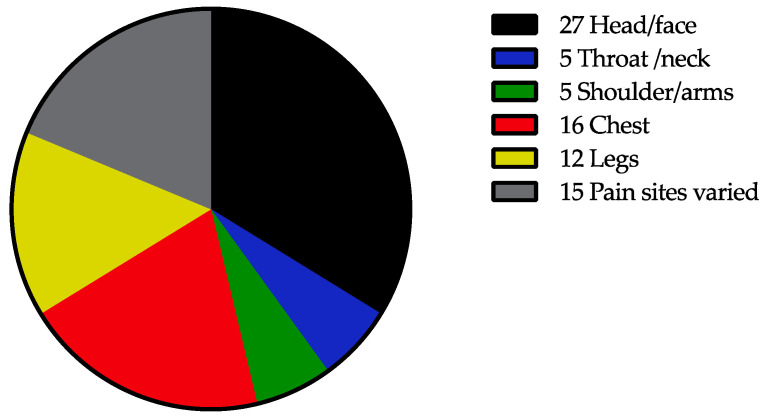
Number of participants indicating the most painful sites.

The mean value of pain intensity during the last week was 4.4 (standard deviation 2.1, range 0–9) from a maximum score of 10. Six participants reported a pain intensity of 0 during the last week. The data for 36 IASP sites in the body are presented in Table S2. More than 50% of the participants indicated pain in the head/face and throat/neck. Approximately 30%—or even more—indicated pain in the shoulder, front of the chest, spine, and feet (Table S2). The most painful sites according to the questionnaire were the head (27%), chest (16%), legs (12%), or varied (15%) (Figure 1).

### 3.4. The Symptom Questionnaire

**Figure 2:**
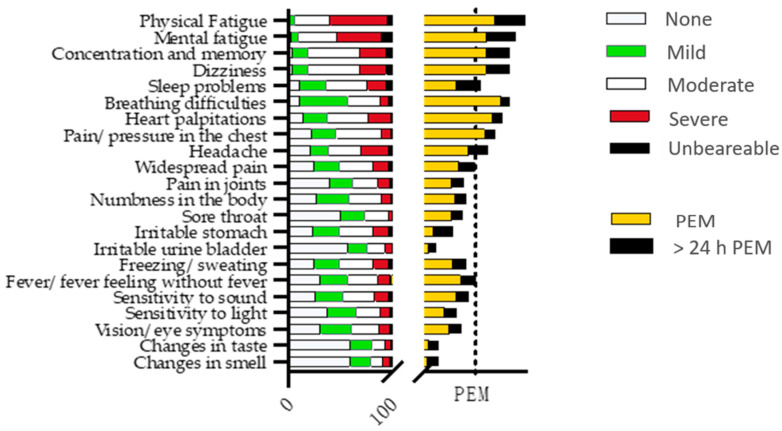
Results of symptom questionnaire are presented as number of participants. Left X-axis indicates symptoms and right X-axis indicates post-exertional malaise (PEM) for every symptom. Both X-axes have magnitude of 100 (*n* = 100); and the dot line on the right X-axis marks 50 for PEM.

Results for multiple symptoms and post-exertional malaise (PEM) are presented in Figure 2, showing grades for multiple symptoms as well as PEM for each symptom. PEM was divided into PEM directly after an exertion and PEM remaining longer than 24 h. Fatigue and cognitive problems were the most enhanced symptoms regarding the level of severity and PEM. More than half of the participants scored physical fatigue as severe and increasing after an effort (PEM). Nearly 30% of the participants reported that exacerbation of fatigue after exertion remained longer than 24 h. Breathing difficulties and heart palpitations were other symptoms, mostly scored as mild or moderate but exaggerated by exertion in over half of the participants (Figure 2). Pain problems were scored by more than 75% of the participants, mostly as moderate, and increasing after exertion, especially in the chest and head (59% and 43%, respectively). Symptoms regarding “irritable stomach” were also reported by more than 75% of the participants, but these were less affected by exertion. Increased nervous system response to stimuli such as sound and light hypersensitivity as well as vision problems were also found in more than half of the participants and were scored mostly as light or moderate, exaggerating after exertion in 20–32% of the participants.

### 3.5. EQ5D, MFI-20, HADS, PHQ-9, GAD-7, and ISI

Self-scored questionnaires showed a low quality of life with a mean value 0.51 (1 is normal), and 99% of participants scored abnormal values for EQ5D. EQ5D VAS was low with a mean value of 42.6 and was abnormal for all participants (Table 2). The category “Bodily Pain” in SF-36 was low with a mean value of 46 and was abnormal in 84% of participants. Abnormal fatigue values were found in 96–100% of the participants according to MFI-20, except for Reduced Motivation (78%) (Table 2). For HADS, the mean values for anxiety and depression were 8 and 8.7, respectively. Depression scores were increased in 28% and 56% of the participants according to HADS and PHQ-9, respectively. Anxiety scores were increased in 14% and 20% of the participants according to HADS and GAD-7, respectively (Table 2). The ISI with a mean value of almost 13 was pathologically increased in 34% of participants (Table 2).

### 3.6. Widespread Pain and Estimation of Fibromyalgia Diagnosis According to the 2016 Criteria and Subgroup Analysis

Table 3 presents the results of 50 participants who had widespread pain in 4/5 regions based on calculations from IASP 36 pain sites. Among them, 30 participants indicated that they were healthy before their infection. When adding the Symptom Severity Scale (re-calculated from the symptom questionnaire) and the Widespread Pain Index (re-calculated from IASP 36 pain sites), 40 participants fulfilled the 2016 criteria for fibromyalgia. Among those, 22 indicated that they were healthy before their infection. The mean pain intensity for those 50 participants having generalised pain was 5.16 (SD 1.7, range 1–9).

To understand the relationship between comorbidities before COVID-19 infection and pain problems and/or medication after COVID-19 infection, subgroup analysis was performed regarding “healthy” vs. “unhealthy”, the number of total comorbidities, and the number of total medications. The results indicate that the number of total medications in the “unhealthy” subgroup was higher than that of the “healthy” subgroup. No difference was found regarding pain medication in subgroup analysis of “healthy” vs. “unhealthy” (Table 4). There was a tendency towards a more frequent estimation of fibromyalgia diagnosis among the “unhealthy” than in the “healthy” subgroup (*p* = 0.08, *n* = 100, Chi-square test). The total number of comorbidities before COVID-19 was low (median = 0, range = 0–5, *n* = 100). Of those who were estimated to have generalised pain and fibromyalgia, the number of comorbidities was statistically higher (*p* = 0.031 and *p* = 0.027, respectively, Mann–Whitney U-test, *n* = 100) (Table 4).

## 4. Discussion

The results of this study confirm the symptoms of post-COVID-19 condition with major symptoms such as fatigue, cognitive/sleep problems, and cardiopulmonary symptoms, especially regarding exacerbation after exertion and persisting longer than 24 h. This phenomenon is known as PEM and is one of the key symptoms in another disabling condition, Myalgic Encephalomyelitis/Chronic Fatigue Syndrome (ME/CFS) [26]. The findings on fatigue and breathing difficulties as major symptoms are supported by a meta-analysis, including 15,244 hospitalised and 9,011 non-hospitalised patients reporting that fatigue and dyspnoea were found among 60% of the pooled population and were the most prevalent post-COVID-19 symptoms after 60 or 90 days or even later after the acute infection [27]. Overall, low health-related quality of life was reported by participants according to EQ5D and SF-36 (bodily pain). Nevertheless, pain problems might still be listed after these most disabling symptoms, confirming that several body functions are impaired and demonstrating the complexity of post-COVID-19 syndrome [14]. The results of the present study show that pain is one of the symptoms in post-COVID-19 syndrome, though not a dominating concern according to self-scored questionnaires.

Recently, Fernandez-de-las-Penas et al. [11] presented a systemic review and meta-analysis of over 14,000 hospitalised and 11,000 non-hospitalised COVID-19 patients. Their results indicate that musculoskeletal and joint pain varied between 4.6% and 23.6% at different follow-up periods during the first year of infection, most frequently at the onset of infection. In the present study, pain was reported by 94% of the population, although pain intensity during the last week was not so high at 4.6 on a 10-point scale when excluding pain-free participants. Pain problems were found mostly in the head/face, chest, spine, and extremities or as migrating pain. Thus, musculoskeletal pain and headache (probably of neuropathic origin) are reported to dominate, although even visceral pain could be suspected (IBS-like symptoms and irritable urinary bladder). In practice, this means that different (new) types of pain might be seen in the same patient suffering from post-COVID-19 syndrome. Although the pain intensity was moderate, many participants who were healthy before infection developed pain, which might be considered a risk factor for chronic pain if no action is taken.

Approximately 75% of our participants reported generalised pain according to the symptom questionnaire. Recalculation of the 36 IASP pain sites and the symptoms from the questionnaires indicated that 50% of the participants had generalised pain and 40% fulfilled the 2016 criteria for fibromyalgia. Among those 40 participants fulfilling the 2016 fibromyalgia criteria, 23 were healthy before their COVID-19 infection. Subgroup analyses indicated that those with comorbidities before COVID-19 infection consumed more medications after infection and tended to develop widespread pain/fibromyalgia according to the 2016 criteria more often compared to “healthy” participants. Those estimated to suffer from widespread pain and fibromyalgia also consumed a higher total number of medications (results not presented). Therefore, one might speculate that comorbidities themselves are a risk factor for the development of pain problems in post-COVID-19 syndrome. At the same time, the total number of medications might simply reflect an increased range of comorbidities following COVID-19 infections. Interestingly, the number of pain medications did not differ between the “healthy” vs. “unhealthy” groups. This might be due to insufficient pain management by (prescribed) medication and/or the important role of comorbidities by themselves in pain development because medication for asthma, hypothyroidism, and psychiatric disorders differed between “healthy” and “unhealthy” participants. An increased number of medications against somatic disorders indicates that not only psychiatric comorbidities matter in the development of pain. At the same time, it is important to note that abnormal values on psychiatric scales were found in fewer than 1/3 of the cohort, except for PHQ-9. PHQ-9 is a depression scale that also includes questions about sleep difficulties, fatigue/fatigability, and cognitive failures—the core symptoms in post-COVID-19 syndrome. Therefore, one should be cautious when interpreting the results of the PHQ-9 alone in post-COVID-19 syndrome for evaluating the risk of depression. The results of the present study might therefore indicate that somatic disorders do have an impact on the development of pain, at least in post-COVID-19 syndrome, since a majority of the participants indicated that they had been healthy before the COVID-19 infection. The impact of somatic comorbidities should be further carefully studied to understand pain pathways in post-COVID-19 syndrome. However, comorbidities have not been well investigated in chronic pain conditions, except for psychiatric ones. Recently, it has been reported that there is an association between rheumatic and psychiatric disorders with fibromyalgia [28] as well as between psychiatric and chronic pain comorbidities with fibromyalgia [29]. However, to our knowledge there is a lack of studies regarding somatic comorbidities and the total number of pharmacological medications in pain disorders, including fibromyalgia.

A recent meta-analysis reported an approximately 10% prevalence of widespread pain in general populations [30]. Gender (women), age (middle aged and older) [30], and lower socioeconomic status [31] are known risk factors. In the present study, pain symptoms were found in socioeconomically wealthy middle-aged women, which is not in line with the previous studies on widespread pain conditions, including fibromyalgia. However, the results of the present study are in line with a previous report on 100 participants with post-COVID-19 syndrome after mild infection participating in a rehabilitation programme [32]. The latter report presents a cohort consisting of middle-aged participants, predominantly females (68%), 75% non-hospitalised, and having similar rates of comorbidities for asthma, hypertension, anxiety/depression, etc., except for diabetes mellitus [32]. It is supposed that two types of post-COVID-19 syndrome might develop after SARS-CoV-2, one after severe initial infection with hospitalisation, predominantly in males [5], and the other after mild initial infection mainly without hospitalisation, predominantly in females [32]. The incidence of post-COVID-19 syndrome is estimated at 10–35% and is higher for hospitalised patients [33]. Therefore, pain disorders in hospitalised patients with post-COVID-19 syndrome may be different when compared to patients after a mild initial infection.

Limitations: This study includes a limited population with post-COVID-19 syndrome after mild infection who were motivated to participate in an online rehabilitation study. The inclusion criterion of at least 50% decreased functioning and activity levels was chosen for the rehabilitation purpose and limits the generalisability of post-COVID-19 syndrome after a mild COVID-19 infection. Generalised pain and fibromyalgia diagnosis, according to the 2016 criteria, were estimated by answers to survey questions, not as direct answers to face-to-face questions. For example, the symptom “cramps in the lower abdomen” was not included in the calculation of fibromyalgia diagnosis, therefore creating a negative bias. Gender perspective was not studied due to the smaller number of male participants. The online collection of data in the present study might have affected the study population because middle-aged women with higher education predominated. The explanation for this overrepresentation might also depend on the fact that these participants might simply be more familiar with social media. On the other hand, it cannot be excluded that non-hospitalised women are more likely to be affected by post-COVID-19 syndrome after a milder form of infection [32]. Therefore, further studies are needed to clarify the role of gender in post-COVID-19 syndrome after mild infection. In the next step, data on pharmacological treatment and comorbidities before and after SARS-CoV-2 should be gathered to understand the interplay between new symptoms, comorbidities, and medication.

Strengths: Face-to-face meetings during the rehabilitation programme indicated that participants fulfilled the criteria for post-COVID-19 condition [7]. Another strength is the estimation of PEM (frequency and degree of PEM for each symptom) when using the symptom questionnaire. In general, there is a lack of instruments to measure PEM. Another strength of the study is the inclusion of comorbidities before and pharmacological treatment after SARS-CoV-2. Together with self-scored questions, this helped to create a more detailed clinical picture regarding the post-COVID-19 condition after a mild COVID-19 infection.

## 5. Conclusions

In conclusion, the results of the present study show the complexity of the post-COVID-19 condition, indicating that (new) pain is quite often reported by post-COVID-19 sufferers despite a mild initial infection. However, other symptoms seem to be more disabling than pain, which might delay adequate pain analysis and management as well as referral to a pain specialist, if indicated. Thus, doctors should be aware of pain development, especially in those with comorbidities before their COVID-19 infection.

## Figures and Tables

**Table 1:** Results of comorbidities before COVID-19 and medication after COVID-19 are presented as the number of participants among 100 participants, except for BMI (*n* = 99). Differences between “Healthy” vs. “Unhealthy” before COVID-19 were analysed by Chi-square 2-tailed test, except for BMI (2-sided *t*-test).

Disorders	Disorders before COVID-19	Taking Medication after COVID-19	Taking Medication after COVID-19, Healthy before, *n* = 68	Taking Medication after COVID-19, Unhealthy before, *n* = 32	*p*-Value
Total number of persons with disorders before infection	32				
Cardiovascular disorders	7	26	15	11	*p* = 0.1
Metabolic diseases:	6				
- hypothyroidism - overweight - PCOS	4 1 1	7 1	2 0	5 1	*p* = 0.03 *p* = 0.3
Lung disorders:	11				
- asthma - chronic obstructive lung disease	10 1	29	12	17	*p* < 0.001
Allergies	2	19	11	8	*p* = 0.2
Psychiatric disorders:	12				
- stress-related exhaustion syndrome - anxiety/depression - bipolar disorder - ADHD - PTSD	1 6 2 2 1	23	10	13	*p* = 0.005
Inflammatory disorders:	9	NSAID, biological drugs	9	3	*p* = 0.4
- endometriosis - Crohn’s disease - arthritis/polyarthritis - chronic pain	2 2 2 3	tricyclic/tetracyclic antidepressants opioids antiepileptics paracetamol	9 1 4 1	4 0 2 2	*p* = 0.6 *p* = 0.7 *p* = 0.6 *p* = 0.2
Vitamin deficiency	2				
Sleep disorders:					
- sleep apnoea syndrome	1	17	9	8	*p* = 0.1
Herpes virus	1				
Anaemia	1				
ME/CFS	1				
BMI (mean, standard deviation in kg/m^2^)	26.5, 5.9		25.2, 3.9	29.3, 8.2	*p* = 0.01

Abbreviations: PCOS = Polycystic Ovarian Syndrome; ADHD = Attention-Deficit/Hyperactivity Disorder; PTSD = Post-Traumatic Stress Disorder; ME/CFS = Myalgic Encephalomyelitis/Chronic Fatigue Syndrome; BMI = Body Mass Index.

**Table 2:** Parameters for health-related quality of life (EQ5D), bodily pain (SF-36), fatigue (MFI-20), emotional status (HADS, PHQ-9, and GAD-7), and sleep (ISI) shown as the mean, standard deviation, and range and as the number of participants with abnormal values. *n* = 100, except for GAD-7, *n* = 97.

Questionnaires	Mean, SD, and Range	Number of Persons with Abnormal Values
EQ5D index	0.51 (0.2) 0.14–1.00	99
EQ5D VAS	42.6 (19.5) 10–83	100
Bodily Pain SF-36	46 (23) 10–100	84
MFI-20 General fatigue	18.5 (2.2) 8–20	98
MFI-20 Physical fatigue	18.1 (2.4) 7–20	99
MFI-20 Reduced activity	17.4 (3.0) 5–20	97
MFI-20 Reduced motivation	11.7 (3.7) 4–20	78
MFI-20 Mental fatigue	14.7 (3.7) 4–2	96
HADS Anxiety	8.0 (3.2) 2–21	14
HADS Depression	8.7 (4.1) 0–19	28
PHQ-9	12.7 (6.1) 0–28	56
GAD-7	5.2 (4.6) 0–19	20
Insomnia Severity Index	12.7 (6.1) 0–28	34

Abbreviations: EQ5D = European Quality of Life Instrument*;* SF-36 = Short Form 36; HADS = Hospital Anxiety and Depression Scale; PHQ-9 (Patient Health Questionnaire); GAD-7 = (Generalised Anxiety Disorder), ISI = Insomnia Severity Index. Pathological values: EQ5D index ≥ 0.8, EQ5D VAS ≥ 85; Bodily Pain SF-36 ≥ 73; HADS ≥ 11 (both anxiety and depression), PHQ-9 ≥ 10, GAD-7 ≥ 10, and ISI ≥ 15.

**Table 3:** Results of the Widespread Pain Index and Symptom Severity Scale regarding the diagnosis of fibromyalgia according to the 2016 criteria. Results are for 50 participants with generalised pain in 4/5 regions.

Widespread Pain Index and Number of Participants (Max 19 Points)	Symptom Severity Scale (Max 12 Points)	Fibromyalgia Diagnosis, Number of Participants
0 (*n* = 1)	3	0
3 (*n* = 1)	4	0
4 (*n* = 1	11	1
5 (*n* = 3)	7–8	0
6 (*n* = 8)	5–10	3
7 (*n* = 8)	8–11	8
8 (*n* = 3)	7–9	3
9 (*n* = 6)	8–11	6
10 (*n* = 5)	9–12	5
11 (*n* = 5)	8–12	5
12 (*n* = 1)	11	1
13 (*n* = 1)	10	1
14 (*n* = 1)	12	1
15 (*n* = 3)	7–10	3
16 (*n* = 2)	10–12	2
19 (*n* = 1)	8	1

**Table 4:** Results for health status (“Healthy” vs. ”Unhealthy”) as well as total number of comorbidities before COVID-19 in comparison with drugs and pain characteristics after COVID-19. The Mann–Whitney U-test (M-W) and Chi-square 2-sided test were used when appropriate.

	Pain Drugs After COVID-19, Median and Range	Total Drugs After COVID-19, Median and Range	No Generalised Pain After COVID-19, *n* = 50	Generalised Pain After COVID-19, *n* = 50	Comparison “No Generalised Pain” vs. “Generalised Pain”	No Fibromyalgia After COVID-19, *n* = 60	Fibromyalgia After COVID-19, *n* = 40	Comparison “No Fibromyalgia” vs. “Fibromyalgia”
*n* = 100								
Healthy before COVID-19, *n* = 68	0 (0–6)	1 (0–9)	38	30		45	23	
Unhealthy, *n* = 32	1 (0–5)	2 (0–7)	12	20		15	17	
Comparison “Healthy” vs. “Unhealthy”	*p* = 0.13 M-W	*p* < 0.001 M-W		*p* = 0.13 Chi-square			*p* = 0.082 Chi-square	
Total comorbidities before COVID-19, median and range			0 (0–2)	0 (0–5)	*p* = 0.031 M-W	0 (0–4)	0 (0–5)	*p* = 0.027 M-W

## Data Availability

The data that support the findings of this study are available from the **c**orresponding author (I.B.L.) upon reasonable request.

## Supplementary Materials

The following are available online at https://www.mdpi.com/article/10.3390/jcm11030771/s1, Supplementary Material S1; Table S1: Background data of 100 participants; Table S2: Results of 36 IASP painful sites (multiple choices allowed) and the most painful sites.

## References

[1] Wu Y., Xu X., Chen Z., Duan J., Hashimoto K., Yang L., Liu C., Yang C. (2020). Nervous system involvement after infection with COVID-19 and other coronaviruses. Brain Behav. Immun..

[2] Yachou Y., El Idrissi A., Belapasov V., Ait Benali S. (2020). Neuroinvasion, neurotropic, and neuroinflammatory events of SARS-CoV-2: Understanding the neurological manifestations in COVID-19 patients. Neurol. Sci..

[3] Masi P., Hekimian G., Lejeune M., Chommeloux J., Desnos C., Pineton De Chambrun M., Martin-Toutain I., Nieszkowska A., Lebreton G., Brechot N. (2020). Systemic Inflammatory Response Syndrome Is a Major Contributor to COVID-19-Associated Coagulopathy: Insights From a Prospective, Single-Center Cohort Study. Circulation.

[4] Group C.-I. (2021). Clinical characteristics and day-90 outcomes of 4244 critically ill adults with COVID-19: A prospective cohort study. Intensive Care Med..

[5] Raveendran A.V., Jayadevan R., Sashidharan S. (2021). Long COVID: An overview. Diabetes Metab Syndr..

[6] Lambert N. (2020). Covid-19 “Long Hauler” Symptom Survey Report. https://dig.abclocal.go.com/wls/documents/2020/072720-wls-covid-symptom-study-doc.pdf.

[7] World Health Organization (2021). A Clinical Case Definition of Post COVID-19 Condition by a Delphi Consensus. https://www.who.int/publications/i/item/WHO-2019-nCoV-Post_COVID-19_condition-Clinical_case_definition-2021.1.

[8] Carfi A., Bernabei R., Landi F., Gemelli Against COVID-19 Post-Acute Care Study Group (2020). Persistent Symptoms in Patients After Acute COVID-19. JAMA.

[9] Huang C., Huang L., Wang Y., Li X., Ren L., Gu X., Liang K., Li G., Min L., Xing Z. (2021). 6-month consequences of COVID-19 in patients discharged from hospital: A cohort study. Lancet.

[10] Townsend L., Dyer A.H., Jones K., Dunne J., Mooney A., Gaffney F., O’Connor L., Leavy D., O’Brien K., Dowds J. (2020). Persistent fatigue following SARS-CoV-2 infection is common and independent of severity of initial infection. PLoS ONE.

[11] Weng L.M., Su X., Wang X.Q. (2021). Pain Symptoms in Patients with Coronavirus Disease (COVID-19): A Literature Review. J. Pain Res..

[12] Fernandez-de-Las-Penas C., Navarro-Santana M., Gomez-Mayordomo V., Cuadrado M.L., Garcia-Azorin D., Arendt-Nielsen L., Plaza-Manzano G. (2021). Headache as an acute and post-COVID-19 symptom in COVID-19 survivors: A meta-analysis of the current literature. Eur. J. Neurol..

[13] Karaarslan F., Demircioglu Guneri F., Kardes S. (2021). Postdischarge rheumatic and musculoskeletal symptoms following hospitalization for COVID-19: Prospective follow-up by phone interviews. Rheumatol. Int..

[14] Norrefalk J.R., Borg K., Bileviciute-Ljungar I. (2021). Self-scored impairments in functioning and disability in post-COVID syndrome following mild COVID-19 infection. J. Rehabil. Med..

[15] Szende A., Janssen B., Cabases J. (2014). Self-Reported Population Helath: An International Perspective Based on EQ-5D.

[16] Sullivan M., Karlsson J. (1998). The Swedish SF-36 Health Survey III. Evaluation of criterion-based validity: Results from normative population. J. Clin. Epidemiol..

[17] Sullivan M., Karlsson J., Ware J.E. (1995). The Swedish SF-36 Health Survey—I. Evaluation of data quality, scaling assumptions, reliability and construct validity across general populations in Sweden. Soc. Sci. Med..

[18] Ericsson A., Bremell T., Mannerkorpi K. (2013). Usefulness of multiple dimensions of fatigue in fibromyalgia. J. Rehabil. Med..

[19] Lungh Hagelin C., Wengström Y., Runesdotter S., Furst C.-J. (2007). The psychometric properties of the Swedish Multidimentional Ftigue Inventory MFI-20 in four different populations. Acta Oncol..

[20] Zigmont A.S., Snaith R.P. (1983). The Hospital and Anxiety Scale. Acta Psychiatr. Scand..

[21] Kroenke K., Spitzer R.L., Williams J.B.W., Löwe B. (2010). The Patient Health Questionnaire somatic, anxiety, and depressive symptom scales: A systematic review. Gen. Hosp. Psychiatry.

[22] Levis B., Benedetti A., Thombs B.D. (2019). Accuracy of patient health questionnaire-9 (PHQ-9) for screening to detect major depression: Individual participant data meta-analysis. BMJ.

[23] Spitzer R.L., Kroenke K., Williams J.B.W., Löwe B. (2006). A brief measure for assessing generalized anxiety disorder: The GAD-7. Arch. Intern. Med..

[24] Bastien C.H., Vallieres A., Morin C.M. (2001). Validation of the Insomnia Severity Index as an outcome measure for insomnia research. Sleep Med..

[25] Wolfe F., Clauw D.J., Fitzcharles M.A., Goldenberg D.L., Hauser W., Katz R.L., Mease P.J., Russell A.S., Russell I.J., Walitt B. (2016). Revisions to the 2010/2011 fibromyalgia diagnostic criteria. Semin. Arthritis Rheum..

[26] Carruthers B.M., van de Sande M.I., De Meirleir K.L., Klimas N.G., Broderick G., Mitchell T., Staines D., Powles A.C., Speight N., Vallings R. (2011). Myalgic encephalomyelitis: International Consensus Criteria. J. Intern. Med..

[27] Fernandez-de-Las-Penas C., Palacios-Cena D., Gomez-Mayordomo V., Florencio L.L., Cuadrado M.L., Plaza-Manzano G., Navarro-Santana M. (2021). Prevalence of post-COVID-19 symptoms in hospitalized and non-hospitalized COVID-19 survivors: A systematic review and meta-analysis. Eur. J. Intern. Med..

[28] Lichtenstein A., Tiosano S., Amital H. (2018). The complexities of fibromyalgia and its comorbidities. Curr. Opin. Rheumatol..

[29] Kleykamp B.A., Ferguson M.C., McNicol E., Bixho I., Arnold L.M., Edwards R.R., Fillingim R., Grol-Prokopczyk H., Turk D.C., Dworkin R.H. (2021). The Prevalence of Psychiatric and Chronic Pain Comorbidities in Fibromyalgia: An ACTTION systematic review. Semin. Arthritis Rheum..

[30] Mansfield K.E., Sim J., Jordan J.L., Jordan K.P. (2016). A systematic review and meta-analysis of the prevalence of chronic widespread pain in the general population. Pain..

[31] Andrews P., Steultjens M., Riskowski J. (2018). Chronic widespread pain prevalence in the general population: A systematic review. Eur. J. Pain..

[32] Vanichkachorn G., Newcomb R., Cowl C.T., Murad M.H., Breeher L., Miller S., Trenary M., Neveau D., Higgins S. (2021). Post-COVID-19 Syndrome (Long Haul Syndrome): Description of a Multidisciplinary Clinic at Mayo Clinic and Characteristics of the Initial Patient Cohort. Mayo Clin. Proc..

[33] Pavli A., Theodoridou M., Maltezou H.C. (2021). Post-COVID Syndrome: Incidence, Clinical Spectrum, and Challenges for Primary Healthcare Professionals. Arch. Med. Res..

