# Supplementary material for: Pain Burden in Post-COVID-19 Syndrome following Mild COVID-19 Infection
Source: J Clin Med. 2022 Jan 31;11(3):771. doi: 10.3390/jcm11030771 (PMC8836662; doi:10.3390/jcm11030771)
Supplement: Supplementary file 1 [file jcm-11-00771-s001.zip › jcm-1542768-supplementary.pdf]

Supplemental Material S1

The following comorbidities were found in the free text: hypertension and other heart disorders, asthma and other lung disorders, hypothyroidism, overweight, PCOS (Polycystic Ovarian Syndrome), allergies, chronic pain, ME/CFS (Myalgic Encephalomyelitis/Chronic Fatigue Syndrome), arthritis, Crohn's disease, endometriosis, ADHD (Attention Deficit Hyperactivity Disorder), anxiety/depression, stress-related exhaustion syndrome, bipolar disorder, PTSD (Post-Traumatic Stress Disorder), sleep apnea syndrome, vitamin deficiency, and anaemia herpes virus infection

Comorbidities were grouped into the following categories: cardiovascular disorders, metabolic disorders, lung disorders, allergies, psychiatric disorders, inflammatory disorders (including pain), sleep disorders, and others. Ongoing medication was indicated in a free text and was grouped as follows: pain modulators, including tricyclic antidepressants and tetracyclic antidepressants (amitriptyline, mirtazapine), antiepileptics (gabapentin, pregabalin), paracetamol, and opioids (tramadol); anti-inflammatory drugs, including nonsteroidal anti-inflammatory drugs (mainly naproxen), corticosteroids, and parental biologically active immunosuppressant (one participant); sleeping aids, including zopiclone, melatonin, promethazine, and hydroxyzine; and psychiatric drugs, including selective serotonin reuptake inhibitors (escitalopram, sertraline, fluoxetine), serotonin-norepinephrine reuptake inhibitors (venlafaxine, duloxetine), and benzodiazepines (oxazepam, alprazolam). Additionally, for cardiovascular disorders, the medications usually included hypertensives or beta blockers, probably for treating tachycardia or postural orthostatic tachycardia syndrome; for hypothyroidism, levothyroxine was used; for asthma, the different asthma inhalers and leukotriene receptor antagonist were used; and for allergies, different antihistamines and/or weak corticosteroids were used in the form of tablets, nasal sprays, or eye drops.

Questionnaires:

The symptom questionnaire

This questionnaire contains questions on multiple symptoms related to COVID infection, graded from "none" to "unbearable" (0–4). Post-exertional malaise (PEM) was asked as questions about deterioration of each symptom after an effort as well as deterioration persisting longer than 24 h. The following 22 symptoms were asked: fatigue in the body; mental fatigue; concentration and memory difficulties; breathing difficulties; pain or unpleasant feeling/pressure in the chest; sore throat or other problems in the throat; heart palpitations; dizziness; sleep problems/changed sleep habits; numbness or strange feelings in the body; changes in taste; changes in smell; irritable stomach/variation in stool; irritable bladder/increased urination; freezing/sweating; fever or feeling of fever without increased temperature; headache; widespread pain; pain in joints; sensitivity to light; sensitivity to sounds; and eye symptoms.

The European quality of life instrument (EQ-5D).

The first part of the instrument defines five dimensions: mobility, self-care, usual activities, pain/discomfort, and anxiety/depression. Using these five dimensions, the instrument calculates an index (EQ-5D Index). The EQ-5D also measures a self-estimation of today's health based on a 100-point scale, a thermometer-like scale (EQ-5D VAS) with

defined end points (high values indicate good health and low values indicate poor health). For a normal Swedish population, EQ5D is 0.85 and the corresponding EQ5D VAS is 85 for people between 35 and 54 years old [15].

The Short-Form 36 (SF-36)

The 36 items in the questionnaire are grouped into 8 subscale scores: physical functioning, role limitations caused by physical problems, bodily pain, general health, energy/vitality, social functioning, role limitations caused by emotional problems, and mental health [16,17].

The Swedish version of the Multidimensional Fatigue Inventory (MFI-20)

It contains 20 statements for which the subject must indicate on a five-point scale the extent to which the statement applies to him or her and covers five dimensions of fatigue: general fatigue, physical fatigue, mental fatigue, reduced motivation, and reduced activity. The statements refer to aspects of fatigue experienced during the preceding days. Higher scores indicate a higher degree of fatigue. For each subscale, a total score is calculated by adding up the scores for the four individual items. The scores can range from 4 to 20 for each subscale [18]. Values for the normal population were chosen from the study reporting on 160 participants from the hospital staff as the control group: general fatigue equal to or under 11; physical fatigue equal to or under 8; mental fatigue equal to or under 9; reduced motivation equal to or under 8; and reduced motivation equal to or under 7 [19].

The Hospital Anxiety and Depression scale (HADS)

The entire sum for both HADS anxiety and HADS depression levels ranges from 0 to 21. A score of equal to or more than 11 allows for clinically significant anxiety or depression symptoms to be suspected [21].

The Patient Health Questionnaire-9 (PHQ-9)

This nine-item questionnaire is designed to assess depressive symptom severity [22]. Self-report items are rated 0 to 3, and total scores range from 0 to 27. The standard cut-off score for screening to identify possible major depression is 10 or above [23].

The Generalised Anxiety Disorder-7 scale (GAD-7)

This seven-item questionnaire is designed to assess anxiety symptom severity. Self-reported items are rated 0 to 3, and total scores range from 0 to 21. The standard cut-off score for screening to identify possible anxiety disorder is recommended 10 or above [24].

The Insomnia Severity Index (ISI)

This self-scored scale measures insomnia during the past 2 weeks. Seven questions assess difficulties related to sleep (to fall asleep, awakenings during night sleep, early awakening), impact of sleep difficulties on other symptoms and day activities as well as satisfaction with sleep and concern about the sleep. The entire sum ranges between 0 and 28 with every question between 0 and 4 according to the Likert scale. Values between 8 and 14 indicate some problems with sleep, while values equal to or above 15 indicate moderate sleep disturbance and equal to or above 22 indicate clinically significant sleep disturbances [25].

Calculation of generalised pain and fibromyalgia diagnosis according 2016 criteria

Fibromyalgia diagnosis according to 2016 was calculated according to the following criteria: 1) generalised pain in 4 of 5 regions;

2) Widespread Pain Index (WPI) equal to or higher than 7 and Severity Symptom Scale (SSS) equal to or higher than 5 or WPI equal to or higher than 4–6 and SSS equal to or higher than 9 [26]. Generalised pain in 4 of 5 regions was calculated by using IASP 36 pain sites and defining regions: 4 quadrants and axial. WPI was also calculated by IASP 36 pain sites as follows: upper regions: jaw = "head/face"; shoulder girdle = "shoulder"; upper arm = "upper arm"; lower arm = "forearm". Lower regions: hip = "hip and/or seat"; upper leg = "thigh"; lower leg = "lower leg". Axial region: neck = "throat and/or neck"; upper back = "thoracic spine" (left or right); lower back = "low back" (left or right); chest = "front of the chest" (left or right); abdomen = "abdomen" (left or right). For Severity Symptom Scale (SSS), fatigue, waking unrefreshed/sleep difficulties, and cognitive problems during the last week were calculated by using corresponding questions in the symptom questionnaire and grading 0–3 (0 = 0, 1 = 1, 2 = 2, and 3 = 3 or 4). The symptom questionnaire was also used to identify the presence of headaches (0 or 1); a HADS equal to or higher than 11 was considered a positive score for depression (0 or 1). "Cramps in the lower abdomen" was not identified by any questionnaire and therefore not included in the analysis.

Table S1. Background data of 100 participants.

Parameters	Number of Persons/Percentage
Age (years in mean, standard deviation, and range)	44.5, 10.6, 20–66
Gender	
Female	82
Male	18
Place of birth:	
Sweden	88<br>12
Outside Sweden	
Marital status:	
Married	49
Having partner	24
Single	27
Having children:	73
Yes	27
No	
Living circumstances:	
Condominium	17
Own house	53
Rental house	22
Inherent	1
Other	7
Education:	

	1
Primary (< 9 years)	31
	61
Secondary (10–12 years)	7
Higher (> 12 years)	
Other education	
Working right now	56
Employed	81
Jobseekers	7
Studying	6
No gainful employment	6
	3
	7
Financial security:	2
Sick-leave 25%	26
Sick-leave 50%	3
Sick-leave 75%	
Sick-leave 100%	10
Disability pension 50%	
Disability pension 100%	3
Unemployment benefits 100%	
Student aid 100%	1
Social security contribution	
Other	4
	2

Table S2. Results of 36 IASP painful sites (multiple choices allowed) and the most painful sites.

Sites	Left Side, Painful	Right Side, Painful	Most Painful Site in the Body
Head and/or face	59	62	27
Throat and/or neck	54	52	5
Shoulder	42	34	5 (shoulder and/or arms)
Upper arm	25	20	
Elbow	7	9	
Forearm	17	19	
Hand	26	23	
Front of the chest	44	33	16 (chest)
Flank of the chest	25	16	
Abdomen	20	22	2
Sexual organs and crotch	5	5	
Thoracic spine	36	39	3
Low back	29	31	5
Hip and/or buttocks	23	24	3
Thigh	18	19	12 (legs)
Knee	12	11	
Lower legs	24	24	

Foot	29	30	
No pain	7	7	
Pain sites varied			15
